# Brown Macroalgae *Sargassum cristaefolium* Extract Inhibits Melanin Production and Cellular Oxygen Stress in B16F10 Melanoma Cells

**DOI:** 10.3390/molecules27238585

**Published:** 2022-12-05

**Authors:** Eka Sunarwidhi Prasedya, Hasriaton Padmi, Bq Tri Khairina Ilhami, Ni Wayan Riyani Martyasari, Anggit Listyacahyani Sunarwidhi, Sri Widyastuti, Miski Aghnia Khairinisa, Nunik Cokrowati, Erika Ernawati Simangunsong, Andri Frediansyah

**Affiliations:** 1 Bioscience and Biotechnology Research Centre, Faculty of Mathematics and Natural Sciences, University of Mataram, Mataram 83126, Indonesia; 2 Department of Biology, Faculty of Mathematics and Natural Science, University of Mataram, Mataram 83126, Indonesia; 3 Department of Pharmacy, Faculty of Medicine, University of Mataram, Mataram 83126, Indonesia; 4 Faculty of Food Technology and Agroindustry, University of Mataram, Mataram 83126, Indonesia; 5 Department of Pharmacology and Clinical Pharmacy, Faculty of Pharmacy, Universitas Padjadjaran, Bandung 45363, Indonesia; 6 Aquaculture Program, Faculty of Agriculture, University of Mataram, Mataram 83127, Indonesia; 7 PT Pavettia Nuansa Alami, Purwakarta 41111, Indonesia; 8 Research Center for Food Technology and Processing (PRTPP), National Research and Innovation Agency (BRIN), Wonosari 55861, Indonesia

**Keywords:** macroalgae, melanin, melanoma, *Sargassum*, tyrosinase

## Abstract

The brown macroalgae *Sargassum* has been reported for its anti-UV and photoprotective potential for industrial applications. This study evaluated the melanin inhibition activity of *Sargassum cristaefolium* (SCE) ethanol extract. Melanogenesis inhibition by SCE was assessed in vitro with B16-F10 melanoma cell models and in silico against melanin regulatory proteins Tyrosinase (TYR) and Melanocortin 1 Receptor (MC1R). The regulatory properties evaluated were the melanin content, intracellular tyrosinase activity and cellular antioxidant activities. In addition, the bioactive compounds detected in SCE were subjected to molecular docking against TYR and MC1R. Based on the results, 150 µg/mL SCE effectively inhibited the production of melanin content and intracellular tyrosinase activity. Cellular tyrosinase activity was reduced by SCE-treated cells in a concentration-dependent manner. The results were comparable to the standard tyrosinase inhibitor kojic acid. In addition, SCE effectively decreased the intracellular reactive oxygen species (ROS) levels in B16-F10 cells. The antioxidant properties may also contribute to the inhibition of melanogenesis. In addition, LCMS UHPLC-HR-ESI-MS profiling detected 33 major compounds. The results based on in silico study revealed that the bioactive compound putative kaurenoic acid showed a strong binding affinity against TYR (−6.5 kcal/mol) and MC1R (−8.6 kcal/mol). However, further molecular analyses are needed to confirm the mechanism of SCE on melanin inhibition. Nevertheless, SCE is proposed as an anti-melanogenic and antioxidant agent, which could be further developed into cosmetic skin care products.

## 1. Introduction

The process of melanin synthesis is known as melanogenesis, which is performed by melanocytes presented among the basal cells of the epidermis [1]. Melanocytes produce melanin in response to ultraviolet (UV) irradiation as a defense mechanism [2]. However, the excessive production of melanin can cause melanoma skin cancer. Increased exposure to UV irradiation is reported to be associated with more than 60% of melanoma cases [3]. Hence, the development of an effective skin UV protection products should also take melanin inhibition activity into consideration. 

Marine macroalgae have been extensively reported to exhibit bioactive compounds with a wide range of biological activities, including UV photoprotective, antioxidant, anticancer and immunomodulatory activity [4,5,6,7,8,9]. Most marine macroalgae are found to inhabit the intertidal zone with an average distance of 3–10 m from the shore [10]. Therefore, to adapt and survive in these extreme conditions, macroalgae must develop a defense mechanism by producing bioactive compounds that can minimize the photodamage induced by excessive UV irradiation. 

Various bioactive compounds in marine macroalgae have been reported to have interesting photoprotective activity, such as mycrosporine-like amino acids (MAAs), sulphated polysaccharides, carotenoids and polyphenols [11,12,13]. To date, more than 500 marine macroalgae have been reported to exhibit MAAs, including the brown macroalgae *Sargassum* [14]. In our previous work, we demonstrated that *Sargassum cristaefolium* contains the MAA compound palythenic acid. This compound potentially contributes to the photoprotective activity of *S. cristaefolium.* Numerous reports have shown the photoprotective activity of the brown macroalgae *Sargassum* [6,15]. However, there are no reports of *Sargassum cristaefolium* potential on melanogenesis inhibition. 

In this study, the melanin inhibition potential of SCE is evaluated based on the cellular melanin content, tyrosinase inhibition and in silico molecular-docking analyses of SCE bioactive compounds against melanin regulatory proteins Tyrosinase (TYR) and Melanocortin 1 Receptor (MC1R). In addition, the effect of SCE on cellular antioxidants is also evaluated. The process of melanogenesis also involves an increase in reactive oxygen species (ROS), which induces oxidative stress in melanocytes [16]. Hence, the antioxidant and free radical scavenging activity also play important roles in photoprotection against the harmful effects of UV radiation.

## 2. Results and Discussion

Melanin is a pigment produced by melanocytes and it plays a vital role in protecting the skin against ultraviolet (UV) radiation. However, the excessive production and accumulation of melanin could cause severe problems, including skin cancer [17]. A type of skin cancer known as melanoma elicits a substantial increase in melanin pigment production [18]. Melanoma is one of the most aggressive forms of cancer and has a high mortality rate [19]. 

Thus, the investigation of therapeutic agents that could inhibit melanogenesis is essential for skin depigmentation or lightning treatments. In addition, the currently reported anti-melanogenic agents, such as hydroquinone, kojic acid and arbutin, sometimes produce side effects, including skin irritation and cell toxicity [20,21,22]. Hence, finding a potent natural product with melanogenesis-inhibiting activity with few side effects is necessary. 

### 2.1. Effects of SCE on B16-F10 Melanoma Cell Viability

**Figure 1:**
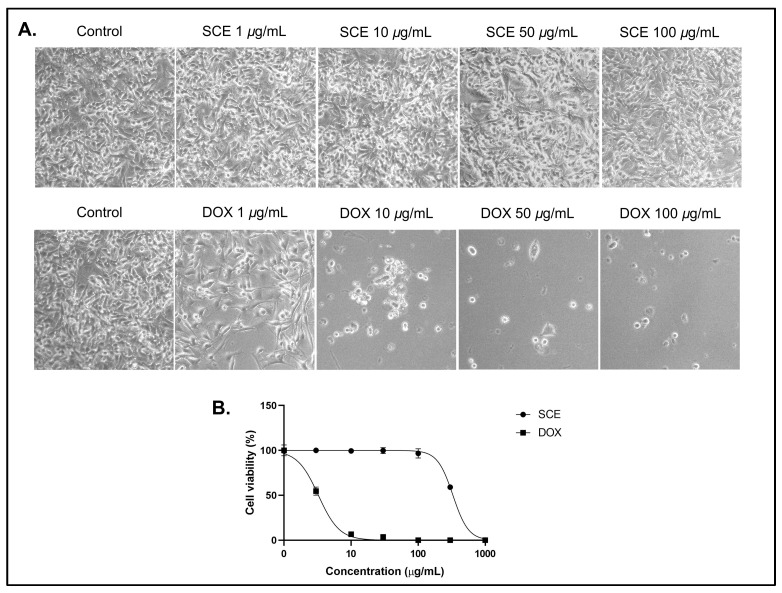
Cytotoxicity assay of SCE against B16-F10 cells for 48 h. (**A**) Morphological observations under a bright field microscope. (**B**) A dose–response curve showing no cytotoxic activity of SCE at the tested concentrations. The results are represented as the means of three independent experiments (SEM ± SD).

To examine the cytotoxic activity of SCE, the B16-F10 melanoma cells were exposed to the SCE extract at concentrations between 1 and 100 µg/mL for 48 h. The SCE-treated cells showed no morphological changes at the tested concentrations (Figure 1A). The anthracycline doxorubicin (DOX) was used as the positive control. Treatment with SCE showed no cytotoxicity (IC_50_ > 100 µg/mL) at the tested concentrations (Figure 1B). 

The positive control DOX was shown to affect the cell morphology at concentrations below 10 µg/mL (IC_50_ = 3.25 ± 0.52 µg/mL). Extracts with IC_50_ greater than 100 µg/mL are considered cytotoxic ineffective extracts [23,24]. A previous report demonstrated that *Sargassum* sp. extract showed low cytotoxicity against Hep-2 and MCF-7 cancer cells with IC_50_ values above 100 µg/mL. [25] Another report also indicated that *Sargassum anguistifolium* extract showed no cytotoxicity in SH-SY5Y cells and prevented methamphetamine toxic effects in SH-SY5Y cells [26]. However, some reports indicate that brown macroalgae Sargassum could be a valuable source of potential anticancer compounds, such as fucoidan. The polysaccharide fucoidan isolated from *Sargassum* has demonstrated anticancer potential in both in vitro and in vivo studies [27,28]. In some cases, a single compound could be more toxic than the whole extract [29,30].

### 2.2. Cellular Antioxidant Activity of SCE

Reactive Oxygen Species (ROS) have been shown to significantly contribute to excess oxidative damage, which results in disease progression, including melanogenesis [31]. Melanogenesis is a biochemical pathway responsible for melanin synthesis in melanocytes. Excessive ultraviolet (UV) radiation can significantly increase the production of melanin, which causes the cell to become heavily pigmented. This then causes the transformation of melanocytes to melanoma, an extremely aggressive form of skin cancer that can spread to various vital organs, including the brain and lungs [32]. In addition, ROS are also produced in melanomas affected by high UV radiation [33]. 

Hence, reducing intracellular ROS levels is important to inhibit melanogenesis progression. In this study, the cells were exposed to UV-A irradiation to induce B16-F10 melanoma cell models. Cells exposed to UV-A irradiation typically demonstrate an increase in cellular Reactive Oxygen Species (ROS) [34]. The levels of ROS in cells were quantified using the oxidation-sensitive fluorescent probe dichlorofluorescein (DCFH) [35]. 

**Figure 2:**
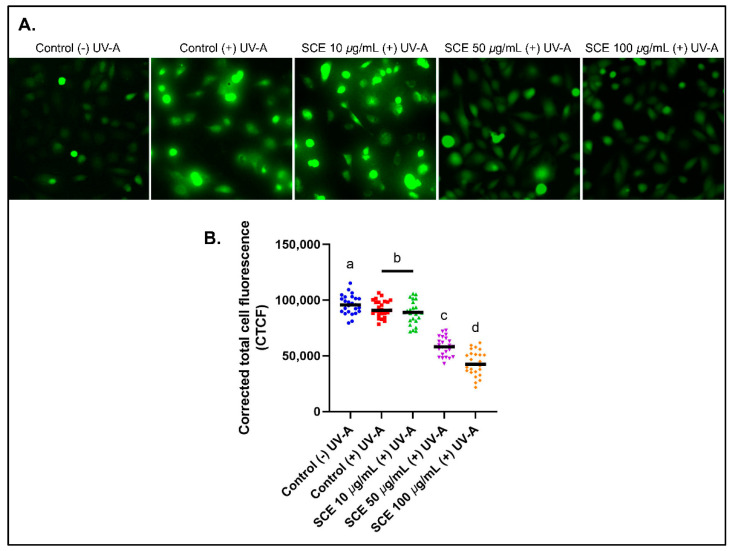
Reactive Oxygen Species (ROS) in B16-F10 cells detected using a H_2_DCFDA fluorescence probe. (**A**) B16-F10 cells emit green fluorescence after UV-A irradiation for 30 min. (**B**) The CTCF values show decreased fluorescence intensity with increased concentrations of SCE in B16-F10 cells, thereby, indicating a decrease in the cellular ROS levels. The results are represented as the means of three independent experiments (SEM ± SD). Different letters indicate a significant difference between treatments (*p* < 0.05).

The esterified form of DCFH (dichlorofluorescein diacetate or DCFH-DA) can penetrate the cell membrane and produce an emission of green fluorescence (Figure 2A). This fluorescence intensity was quantified to determine the cellular ROS levels. In addition, the intracellular ROS levels of SCE-treated B1-F10 cells decreased in a concentration-dependent manner (Figure 2B). A previous study showed that *Sargassum horneri* methanol extract protects C2C12 skeletal muscle cells from oxidative stress [36]. 

Other brown macroalgae, such as *Gongolaria baccata* extract, demonstrated potent cytoprotective activity in Caco-2 cells from oxidative stress induced by tert-butyl hydroperoxide [37]. Regarding brown seaweeds, their cytoprotective effects are possibly due to the presence of various terpenoids, phenols and phlorotannin. Phenols in brown macroalgae, particularly phlorotannins, play roles as chelating agents with reactive oxygen species and consequently prevent cellular oxidative stress and tissue damage [38,39]. 

### 2.3. SCE Effects in B16-F10 Melanin Content

**Figure 3:**
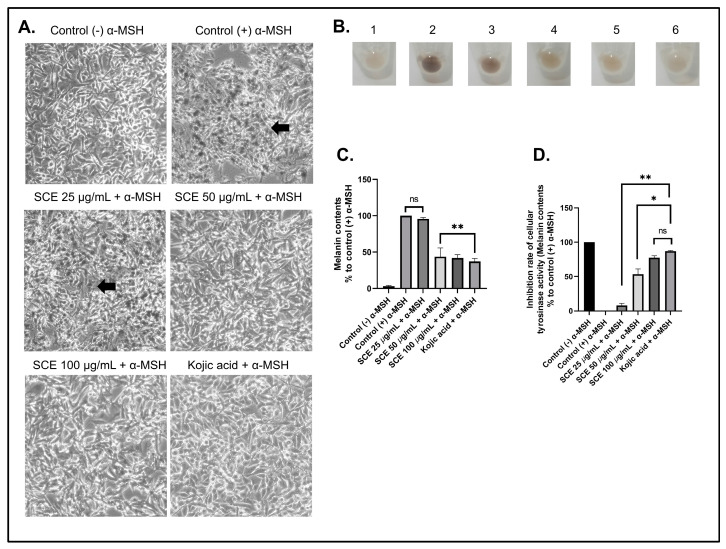
The B16-F10 cells were treated with α-MSH to induce melanin production. (**A**) Morphological observations showing the normal cell morphology of cells treated with α-MSH and SCE. (**B**) Harvested cell pellets of α-MSH-treated cells with SCE. (**C**) Melanin content analyses of α-MSH-treated cells with SCE. (**D**) Tyrosinase activity inhibition in α-MSH-treated cells with SCE. The results are represented as the means of three independent experiments (SEM ± SD). * Indicates significant difference between treatments (*p* < 0.05). ** indicates a highly significant difference between treatments (*p* < 0.01). Black arrows indicate melanin production. ns indicates no significant difference.

To determine the effects of SCE on inhibiting cellular melanin content production, the B16-F10 cells were first treated with the alpha-melanin stimulating hormone (α-MSH) [40]. In the normal physiology process, melanin synthesis is induced by α-MSH secreted by keratinocytes, which would then trigger melanin synthesis through binding with MC1R [41,42]. Figure 3A shows that B16-F10 cells produced melanin after 72 h treated with α-MSH. This production of melanin in B16-F10 cells was inhibited by increasing the concentration of SCE. 

A similar result was also seen in α-MSH-induced B16-F10 cells treated with the positive control kojic acid (100 µM). Furthermore, the B16-F10 cells, which produce high melanin content, would result in a dark brown-colored cell pellet (Figure 3B). This dark brown color decreased in cell pellets treated with SCE and kojic acid. This indicates that SCE showed potent inhibition of the melanin content production. The treatment with SCE above a 50 µg/mL concentration demonstrated a significant decrease in melanin content compared to the control treated with only α-MSH (Figure 3C). In addition, the melanin content in higher concentrations of SCE showed no significant difference compared to kojic acid. 

Kojic acid is a natural metabolite derived from fungi that can inhibit tyrosinase activity, which plays an important role in the synthesis of melanin [43]. Kojic acid showed strong inhibitory activity against the tyrosinase enzyme (Figure 3D). Our results show that increased concentrations of SCE treatment also increased cellular tyrosinase inhibition in B16-F10 cells. 

Moreover, the treatment with 100 µg/mL concentration of SCE resulted in 77.33% tyrosinase activity inhibition. Similar results were also seen in a study on *Sargassum thunbergia,* which showed strong tyrosinase inhibition activity (88.3%) [44]. A previous study that screened 43 indigenous marine algae for tyrosinase inhibitory activity showed that the brown macroalgae *Ecklonia* and *Sargassum* showed potent tyrosinase inhibitory activity comparable to kojic acid [45].

### 2.4. Phytochemical Profling and Molecular Docking of SCE Compounds

To evaluate the potential putative compounds in SCE that possibly contribute to the inhibition of melanogenesis, the extract was subjected to UHPLC-HR-ESI-MS and analyzed using Compound Discoverer 3.2 (Thermo Scientific^TM^, Waltham, MA, USA) with references related to marine-related natural products. Each compound’s putative identity was confirmed using MS1 and MS2. 

Based on the peak area, the highest putative compound found in SCE extract was putatively pheophorbide A of *m*/*z* 593.27496 ([M+H]+), the MS/MS fragmentation pattern corresponding to Chen et al. (2015) [46] with the MS/MS fragmentation of *m*/*z* 431.22223; 445.20157; 533.25433; 547.23254; 593.27496 ([M+H]+), which is also shown in Supplementary Figure S1. This porphyrin derivative was discovered earlier in the brown algae *Saccharina japonica* (former name *Laminaria japonica*), where it inhibited NO generation in LPS-stimulated RAW 264.7 cells [47]. This anti-inflammation component was also discovered in the green algae *Klebsormidium flaccidum* [48].

The putative 2-monoolein of *m*/*z* 357.29940 ([M+H]+) was the second highest putative compound detected in SCE. This compound was previously found and isolated from the brown algae *Ishige sinicola* [49]. Putative eicosapentaenoic acid (C20:5, ω-3) with *m*/*z* 303.23117 ([M+H]+) was the third highest. This acid was previously discovered in the brown alga *Zonaria tournefortii* [50]. The putative kaurenoic acid of *m*/*z* 303.23117 ([M+H]+) was the fourth highest. 

This terpene’s production gene was also discovered in the algae *Laurencia dendroidea* [51]. The putative compound halocynthiaxanthin 3-acetate had the greatest *m*/*z* 641.41821 ([M+H]+), followed by stearidonic acid and α-monopalmitin, which had *m*/*z* 277.21555 and 331.33047 ([M+H]+), respectively. The total of 33 putative compounds that were detected in SCE, including these MS/MS patterns and the corresponding references, are shown in Supplementary Table S1 and Supplementary Figure S1.

A total of 27 compounds from all 33 compounds were selected based on having a molecular weight (MW) below 500 Dalton for effective penetration through the skin layer [52]. The compound kaurenoic acid (C_20_H_30_O_2_) showed the highest binding activity against TYR (−6.5 kcal/mol) and MC1R (−8.6 kcal/mol). This was significantly higher than the positive control kojic acid with the binding affinity of −4.2 kcal/mol for both target proteins TYR and MC1R (Table 1). 

**Figure 4:**
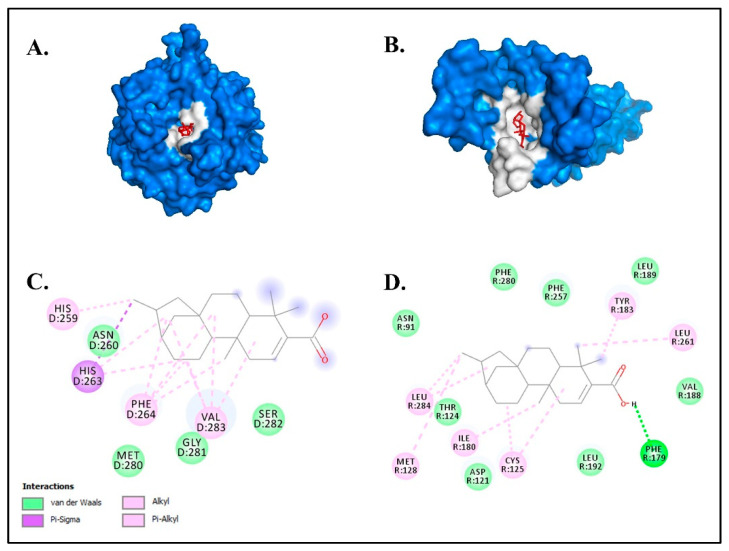
The molecular docking results of kaurenoic acid against the melanogenesis-related proteins (**A**) TYR and (**B**) MC1R. The chemical bond ligand–protein interaction of kaurenoic acid with (**C**) TYR and (**D**) MC1R.

The larger negative values of the total net charge represent a higher binding affinity of the ligand against the target protein [53]. Based on ligand–protein interaction analyses, the compound kaurenoic acid formed a conventional hydrogen and van der Waals bond with TYR and MC1R (Figure 4). 

**Figure 5:**
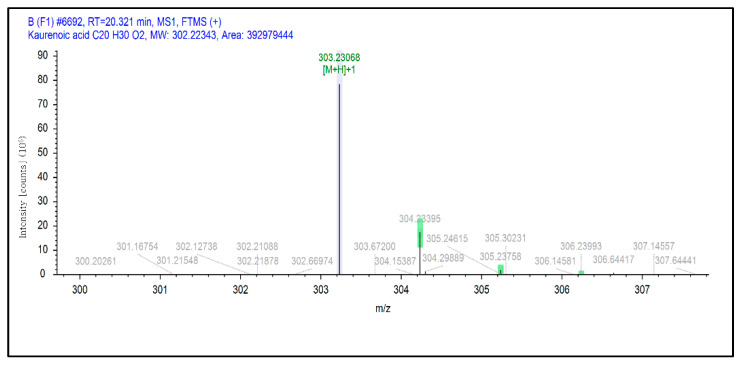
Selected extracted ion chromatogram of kaurenoic acid in SCE.

These two chemical bonds are considered to be significantly strong compared to other chemical bonds, such as metallic, ionic and covalent bonds [54]. Kaurenoic acid has been described as a potential tyrosinase inhibitor in previous studies in various plants [55,56,57,58]. However, this is the first study to report the detection of putative kaurenoic acid in macroalgae or seaweeds (Figure 5).

## 3. Materials and Methods

### 3.1. Chemical and Reagents

Kojic acid, α-MSH, doxorubicin (DOX), 3,4-dihydroxyphenylalanine (L-DOPA), tyrosine, 3-(4,5-dimethyl-2-thiazolyl)-2,5-diphenyltetrazolium bromide (MTT), fluorescent dye CM-H2DCFDA and dimethyl sulfide oxide (DMSO) were purchased from Sigma-Aldrich (St Louis, MO, USA). Organic solvents were purchased from Merck Millipore at HPLC grade (Darmstadt, Germany). Dulbecco’s Modified Eagle’s Medium (DMEM), Fetal Bovine Serum (FBS), trypsin, penicillin and streptomycin were purchased from Thermo Scientific Co. (Shanghai, China).

### 3.2. Plant Materials and Extraction 

The brown macroalgae *Sargassum cristaefolium* was collected from the western coast of Lombok, Batu Layar coast (8°24′11.7396″ S, 116°4′1.9056″ E). The macroalgae samples were identified by referring to an electronic database containing algae taxonomy and nomenclatural information. The collected samples were pre-treated three times by washing with distilled water to remove unwanted debris. Once the samples were clean, they were subjected to the drying process. The drying process was performed at room temperature (24 °C) controlled by an air conditioner. In addition, the samples were also treated with 70% ethanol and 1% fungicide to prevent the growth of microorganisms. Every 24 h, the samples were flipped to avoid moisture accumulation. 

After three days, the samples were transferred into an oven (40 °C) until they reached a constant weight. The resulting dried seaweed biomass was extracted with ethanol (96%) by a maceration process [59]. The dried seaweed biomass was submerged in ethanol (96%) with a ratio of 1:10 *w*/*v* with constant stirring of 100 rpm on a magnetic stirrer. The mixture was then filtered with a cloth every 24 h. The filtrates were combined and subjected to a rotary evaporator (56 °C, 45 rpm and 320 bar) to remove the solvent. The resulting paste was then used as the ethanol extract of *Sargassum cristaefolium* (SCE). The SCE was stored at −20 °C for further analyses. 

### 3.3. Cell Culture

The B16-F10 melanoma cells (ECACC-92101204) were cultured in Dulbecco’s Modified Eagle’s Medium (DMEM; Hyclone, Logan, UT, USA) with 10% fetal bovine serum (FBS) supplemented with penicillin/streptomycin (100 IU/ 50 µg/mL). The cells were kept and grown in a humidified atmosphere containing 5% CO_2_ at 37 °C. 

### 3.4. MTT Cytotoxicity Assay

The cytotoxic activity of SCE was evaluated using an MTT-based assay [60]. The B16-F10 cells were seeded into 96-well plates with a density of 1 × 10^4^ cells/well. After 24 h, the cell culture medium was changed with the treatment medium containing SCE at various concentrations 10–1000 µg/mL). The cells were then further incubated for another 72 h in a humidified CO_2_ incubator. After the treatment, the medium was changed into MTT solution and incubated at 37 °C for 2 h. The MTT solvent DMSO was added to the medium and incubated for 15 min. Finally, the absorbance was measured at 570 nm using a UV-VIS spectrophotometer. The dose–response graft and IC_50_ values were generated with GraphPad software version 9.4.1. 

### 3.5. Determination of Cellular Antioxidant Activity

The cellular antioxidant activity of SCE was determined by labeling cellular ROS levels with fluorescence probe H2-DCFDA [61]. The B16-F10 melanoma cells were seeded at 3.5 × 10^4^ cells/cm^2^ in 60 mm cell-culture dishes. The cells were kept at 37 °C and 5% CO_2_ for 24 h to adhere. The cells were then pre-treated with increasing concentrations of SCE for 1 h prior to UV-A irradiation. The increase in cellular ROS was induced by UV-A irradiation (8 J/ cm^2^) for 30 min [62]. 

After UV-A irradiation, the cells were washed with serum-free medium and analyzed with 25 µM dichlorofluorescein diacetate (DCF-DA) for 30 min at 37 °C in the dark. Intracellular ROS would react with DCF-DA, which emits green fluorescent light. Thus, the cellular ROS levels could be quantified with a fluorescence-inverted microscope (Axio observer Z1, Zeiss, Germany). The fluorescence intensity was determined using the corrected total cell fluorescence (CTCF) equation: CTCF = integrated density—(area of selected cell × mean fluorescence of background readings) [63]. 

### 3.6. Determination of Melanin Content 

The B16-F10 melanoma cells were seeded into 12-well plates with a seeding density of 3 × 10^4^ cells [64]. After 24 h, the cells were injected with alpha-melanocyte-stimulating hormone (α-MSH; 100 nM), followed by the addition of kojic acid or SCE. The treatment was stored in a 37 °C CO_2_ incubator for 72 h. The cells were washed with PBS and lysed with 1 N NaOH for 1 h at 60 °C. The melanin content was determined at 450 nm absorbance and normalized to the total protein content measured by Bradford assay. The morphology of the cells was also documented (Zeiss primo vert, Zeiss, Germany).

### 3.7. Measurement of Cellular Tyrosinase Activity 

The B16-F10 cells were seeded at a density of 10 × 10^4^ cells/well in 12-well cell-culture plates. After 24 h, the cells were treated with SCE or kojic acid for 2 h and then stimulated with 100 nM α-MSH for an additional 72 h. The cells were then washed with PBS and lysed with lysis buffer. The cell lysates were centrifuged at 5000 rpm for 15 min at 4 °C. The enzyme activity was normalized to the protein concentration as determined with the Bradford assay. The reaction of the cellular tyrosinase and L-DOPA solution was performed at 37 °C for 1 h [65]. The production of dopachrome was measured at 490 nm absorbance. Dopachrome is then converted into a synthetic form of melanin [59]. 

### 3.8. UHPLC-HR-ESI-MS Analyses of SCE

The bioactive compounds in SCE were profiled with UHPLC Vanquish Tandem Q Exactive Plus Orbitrap HRMS (Thermo Fisher Scientific, Waltham, MA, USA). Separations were performed on an Accucore C18 column (Thermo Fisher Scientific, 100 mm × 2.1 mm × 1.5 µm) at 30 °C with a flow rate of 0.2 mL/min. We employed a sheath gas flow rate of 15, an auxiliary gas flow rate of 3, a sweep gas flow rate of 0, a spray voltage of 3.80 kV, a capillary temperature of 320 °C, an auxiliary gas heater temperature of 0 °C and an S-lens RF level of 50.0. 

The resolution was set at 70,000 for the entire MS, with an AGC target of 3 × 10^6^ and a maximum IT of 100 ms. In addition, the resolution for dd-MS^2^ was set to 17,500 with an AGC target of 1 × 10^5^ and a maximum IT of 50 ms. Furthermore, the loop count was set to 5, topN was set to 5, the isolation window was 4.0 *m*/*z*, no fixed first mass was set up, and the (N) CE/stepped (N)CE was 18, 35 and 53, with TopN. For the dd setting, the minimum AGC target was 8 × 10^3^ with an intensity threshold of 1.6 × 10^5^, and no apex trigger or charge exclusion was set up. The excluded isotope must be enabled, and the dynamic exclusion time must be set to 10.0 s. 

The mobile phase consisted of water (solvent A) and acetonitrile (solvent B), both acidified with 0.1% formic acid. The gradient was operated as follows: 0.1 min at 5% B, 1–25 min at 5–95% B, 25–28 min at 95% B and 28–30 min at 5% B. Before being injected at a volume of 2.0 µL, the extracted sample was filtered with polytetrafluoroethylene (PTFE) 0.2 µm. The ESI conditions were set to positive over a range of 100–1500 *m*/*z*. [66]. Caffeine was used as a calibrant in the study. The putative compounds were identified using the Compound Discoverer Library version 3.2 and the references.

### 3.9. In-Silico Molecular Docking Analyses of Bioactive Compounds Detected in SCE

The acquired bioactive compounds in SCE were subjected to in silico molecular docking against melanogenesis-related proteins tyrosinase (TYR, PDB ID: 2Y9X) and melanin-concentrating hormone receptor 1 (MC1R, PDB ID: 7F41) [67]. From 33 acquired bioactive compounds from LCMS UHPLC-HR-ESI-MS analyses, 27 compounds were selected as ligands based on molecular weight (<500 Da). 

In addition, these ligand candidates were prepared in 2D form with ChemDraw Ultra 12.0, which was further converted to 3D form with Chem3D Pro 17.0. The protein receptors were prepared with DS BIODIVIA Discovery Studio 2016 v16.1.0 × 64. The grid box was determined by redocking with AutoDockTools-1.5.7 [68]. 

The grid box for the TYR receptor was positioned at the center x, y, z (22.548, 2.483, −93.071) with the size of (Å) x, y, z (40, 40, 40). The grid box for the MC1R receptor was positioned at the center x, y, z (92.688, 80.386, 111.935) with the size of (Å) x, y, z (44, 40, 40). All ligands were subjected to molecular docking with AutoDock Vina v.1.2.0 [69]. The interaction between ligand and receptor was investigated using DS BIOVIA Discovery Studio 2016 v16.1.0 × 64. The visualization between ligand and receptor was generated with PyMOL software v.2.4.1 [70].

### 3.10. Statistical Analyses

All data are shown as the mean ± SD. The data were analyzed using one-way ANOVA. Differences were considered significant if *p* < 0.05. All analyses were performed using GraphPad Prism software for Windows, version 9.4.1.

## 4. Conclusions

In conclusion, in this study, we proposed melanin-inhibiting activity of brown macroalgae *Sargassum cristaefolium,* which can inhibit melanin content and tyrosinase activity. In addition, the ethanol extract of *Sargassum cristaefolium* (SCE) could reduce the oxidative species (ROS) levels in UV-A irradiated cells. Among all compounds in SCE, diterpene kaurenoic acid potentially contributes to SCE melanin-inhibiting activity. 

Based on in silico molecular-docking analyses, the putative kaurenoic acid showed the highest binding affinity for the melanogenesis-related proteins TYR and MC1R. However, the compound needs to be further isolated and characterized in in vitro and in vivo models. Nevertheless, SCE shows potential to be developed into a skin UV-protection agent for cosmetic or medicinal industrial applications. 

## Figures and Tables

**Table 1:** The binding affinity results of kaurenoic acid compared to the standard tyrosinase inhibitor kojic acid.

Compound	Receptor	Binding Affinity (kcal/mol)
Kaurenoic Acid	TYR	−6.5
Kaurenoic Acid	MC1R	−8.6
Kojic Acid	TYR	−4.2
Kojic Acid	MC1R	−4.2

## Data Availability

The data presented in this study are available on request from the corresponding and first author.

## Supplementary Materials

The following supporting information can be downloaded at: https://www.mdpi.com/article/10.3390/molecules27238585/s1, Table S1: Predicted bioactive compound of SCE using untargeted HRMS method, Table S2: Docking result of all ligands with MW < 500 Da against TYR, Table S3: Docking result of all ligands with MW < 500 Da against MC1R, Table S4: Summary of amino acid residues interacting with the ligand kaurenoic acid. Figure S1: Total ion chromatogram of UHPLC-HR-ESI-MS analysis of SCE (A) and blank (B).

## Sample Availability

Samples of the compounds are available from the authors.
