# Supplementary material for: Brown Macroalgae Sargassum cristaefolium Extract Inhibits Melanin Production and Cellular Oxygen Stress in B16F10 Melanoma Cells
Source: Molecules. 2022 Dec 5;27(23):8585. doi: 10.3390/molecules27238585 (PMC9741006; doi:10.3390/molecules27238585)
Supplement: Supplementary file 1 [file molecules-27-08585-s001.zip › molecules-2047029-supplementary.pdf]

SUPPLEMENTARY DATA

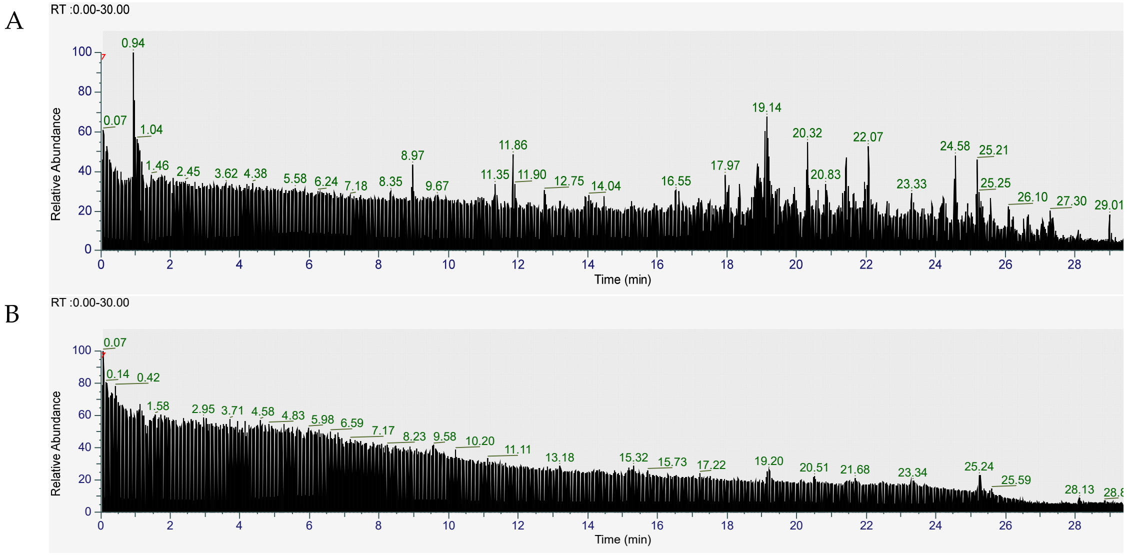

Supplementary Figure S1. Total ion chromatogram of UHPLC-HR-ESI-MS analysis of SCE (A) and blank (B)

Supplementary Table S1. Putative compounds identified from SCE.

No	Putative compound	Formula	MS/MS fragmentation	Annot. Delta Mass [ppm]	Calc. MW	RT [min]	Area	Ref.
1	Acetylcholine	$C_7H_{15}NO_2$	60.08125; 87.04433; 146.11731	-3.48	145.10977	1.083	59650976.75	[1]
2	Adenine	$C_5H_5N_5$	94.03974; 109.0573; 119.03519; 136.06154	-2.52	135.05416	1.106	54016545.93	[2]
3	13S-hydroxyoctadecatrienoic (13-HODE)	$C_{18}H_{30}O_3$	59.01252; 96.95886; 113.09599; 179.14340; 195.13829; 277.21698; 295.22754	-3.82	294.21837	16.727	61663102.62	[3]
4	1-(9Z-hexadecenoyl)-glycero-3-phosphoethanolamine	$C_{21}H_{42}N_7P$	57.07040; 71.08591; 81.07008; 95.08583; 282.27914 452.29254	-4.14	451.26802	17.779	70458077.22	[4]
5	2,3-dihydroxypropyl 12-methyltridecanoate	$C_{17}H_{34}O_4$	57.07036; 95.08575; 137.13200;	-4.73	302.24428	18.789	47036562.18	[5]

			211.20512; 229.2149; 285.24179; 303.22400					
6	Decanoic acid derivative	$C_{29}H_{50}O_4P_2S$	57.07038; 109.11098; 137.13220; 239.23669; 313.27292; 557.29663	-3.05	556.2888	18.891	64080476.4	[6]
7	Stearidonic acid	$C_{18}H_{28}O_2$	55.05470; 79.05457; 93.07009; 107.08565; 121.10113; 135.11662; 149.13228; 163.11133; 179.14270; 235.16904; 277.21555	-3.94	276.20784	18.898	280688522.9	[7,8]
8	AAL Toxin TE2	$C_{27}H_{49}NO_9$	67.05470; 95.08574; 107.08569; 149.13240; 243.21030; 261.22083; 532.34625	-3.3	531.33898	18.980	64724624.36	[9,10]
9	Ebelactone B	$C_{21}H_{36}O_4$	67.05463; 71.08586; 121.10106; 135.11647; 173.13199; 243.21019; 261.22052; 353.26782	-4.65	352.25972	18.982	160256015.1	[11]
10	1-Palmitoyl-2-hydroxy-sn-glycero-3-PE or 16:0 Lyso-PE	$C_{21}H_{44}NO_7P$	62.06050; 127.72014; 318.27266; 394.23932; 454.29254;	-4.71	453.2834	19.416	87112084.83	[12,13]
11	Palythene	$C_{13}H_{20}N_2 O_5$	67.05463; 95.08572; 145.10118; 169.10060; 165.19333; 285.14569	-1.2	284.13814	19.302	125390291.1	[14]
12	α-Eleostearic acid	$C_{18}H_{30}O_2$	67.05467; 81.07021; 95.06574; 121.10110; 149.02316; 243.21049; 279.23135	-4.22	278.22341	19.948	128378549.6	[15-17]
13	1-O-Palmitoyl Glucuronic acid	$C_{22}H_{42} O_7$	67.05466; 147.06503; 165.07561;	-4.32	418.29125	19.953	66238316.76	[18]

			237.22134; 419.29852					
14	2-Arachidonoyl glycerol	$C_{23}H_{38}O_4$	67.055470; 81.979021; 91.05450; 105.0700; 133.10101; 171.11649; 203.17897; 259.24124; 287.23642; 347.25714; 379.28354	-4.72	378.27522	20.287	129418985.2	[19,20]
15	Eicosapentaenoic acid (C20:5, ω-3)	$C_{20}H_{30}O_2$	243.20964; 267.21002; 285.22083; 303.23117	-3.89	302.22341	20.312	401558089.1	[21-24]
16	Farnesylacetone	$C_{18}H_{30}O$	67.050466; 95.08575; 109.10129; 149.13231; 179.17851; 245.22589; 263.23639	-3.39	262.22878	20.316	56312925.5	[25-27]
17	1-linoleoyl glycerol	$C_{21}H_{38}O_4$	67.95456; 71.08592; 95.08575; 109.1037; 135.10110; 179.17865; 245.22588; 253.23636; 337.27295	-4.61	354.27538	20.319	193559408.6	[28]
18	Kaurenoic acid	$C_{20}H_{30}O_2$	67.05464; 91.05445; 131.08536; 145.10091; 169.10088; 257.18930; 304.29907; 303.23117	-1.7	302.23068	20.321	392979444.0	[29]
19	Halocynthiaxanthin 3-acetate	$C_{42}H_{56}O_5$	67.41901; 119.08557; 221.15296; 549.35541; 641.41821	-4.68	640.40978	20.418	371667009.2	[30-32]
20	α-monopalmitin	$C_{19}H_{38}O_4$	58.06561; 91.05446; 95.09582 109.10131; 156.33553; 240.26807; 314.27731 331.33047	-4.3	330.27559	21.443	247963549.9	[33,34]
21	9-octadecenoic acid methyl ester (derivative)	$C_{19}H_{36}O_3$	69.0703; 71.08595; 95.08576;	-3.65	312.26531	21.443	59230522.65	[35]

			137.13202; 157.10048; 181.10132; 197.13181 239.23665; 257.24722; 313.27307					
22	2-Monoolein	$C_{21}H_{40}O_4$	95.0701; 121.10118; 149.13214; 247.24153; 265.25208 283.26316; 357.29940	-4.14	356.29119	22.064	452070448.9	[36,37]
23	Glycidyl oleate	$C_{21}H_{38}O_3$	69.07030; 97.10134; 109.10125 163.14729; 247.24104; 266.25540; 339.28882	-4.34	338.28063	22.064	121668440.7	[38]
24	D-glucitol monooleate	$C_{24}H_{46}O_7$	69.03391; 83.04941; 129.05449; 147.06502; 165.07607; 265. 25244 411.31015; 446.95746	-4.43	446.32237	22.63	133561095	[39]
25	Eicosapentaenoic acid methyl ester	$C_{21}H_{32}O_2$	243.21056; 267.21088; 285.22064; 317.24670	-3.89	316.239	23.34	97976954.15	[40-42]
26	Octadecatrienoic acid	$C_{19}H_{32}O_2$	243.21031; 261.22037; 293.24680	-4.52	292.23891	23.431	67126068.27	[43,44]
27	Farneside B	$C_{24}H_{40}N_2O_8$	67.53648; 91.05457; 105.07009; 134.10388; 157.94377; 185.69495; 485.28665	-1.71	484.27764	24.207	165052165.8	[45]
28	Podecdysone B	$C_{27}H_{42}O_6$	287.23633; 361.27322; 463.30338	-4.88	462.29588	24.226	65408472.9	[46]
29	Protoporphyrin	$C_{34}H_{34}N_4O_4$	67.36295; 131.08536; 431.22322; 504.25067; 563.26521;	-4.81	562.25542	25.014	99755718.43	[47,48]
30	Arachidonic acid	$C_{20}H_{32}O_2$	81.06988; 133.10117; 147.11683; 221.15360; 305.24707;	-4.54	304.23885	25.324	75238762.64	[21]

31	Ponasterone A (Ecdysterone)	$C_{27}H_{44}O_6$	67.05467; 93.07012; 121.10119; 177.16331; 465.31946; 448.31372; 430.31916	-4.51	464.31169	25.573	79557257.27	[49]
32	Pheophorbide A	$C_{35}H_{36}N_4O_5$	67.36158; 141.85011; 268.25776; 431.22223; 445.20157; 533.25433; 547.23254 593.27496	-3.14	592.26671	26.669	454961307.5	[50-52]
33	Methyl pheophorbide a	$C_{36}H_{38}N_4O_5$	67.53841; 547.26935; 607.29083	-3.91	606.28185	29.536	54073422.3	[53]

Supplementary Table S2.

Amino Acid Residues Summary

Compound	Receptor	Amino Acid Residues	Bond Type
Kaurenoic Acid	TYR	HIS D:259, ASN D:260, HIS D: 263, PHE D:264, MET D:280, GLY D:281, VAL D:283, SER D:282	Van der Waals, Pi-Sigma, Alkyl, Pi- Alkyl
Kaurenoic Acid	MC1R	ASN R:91, LEU R:284, MET R:128, THR R:124, ILE R:180, ASP R:121, CYS R:125, LEU R:192, PHE R:179, VAL R:188, LEU R:261, TYR R:183, LEU R:189, PHE R:257, PHE R:280	Van der Waals, Conventional Hydrogen Bond, Alkyl, Pi-Alkyl

Supplementary Table S3.

Docking results of kaurenoic acid with TYR. The grey cells indicate the excluded ligands due to their molecular weight which exceeds 500 Dalton.

No	Putative compound	Formula	Calc. MW	Binding Affinity (kcal/mol)-TYR	Dist from rmsd l.b.	best mode rmsd u.b.
1	Acetylcholine	C7 H15 N O2	145.10977	-3.5	0.000	0.000
2	Adenine	C5 H5 N5	135.05416	-6.0	0.000	0.000

3	13S-hydroxyoctadecatrienoic	C18 H30 O3	294.21837	-3.8	0.000	0.000
4	1-(9Z-hexadecenoyl)-glycero-3-phosphoethanolamine	C21 H42 N O7 P	451.26802	-5.8	0.000	0.000
5	2,3-dihydroxypropyl 12-methyltridecanoate	C17 H34 O4	302.24428	-5.0	0.000	0.000
6	Decanoic acid	C29 H50 O4 P2 S	556.2888			
7	Stearidonic acid	C18 H28 O2	276.20784	-5.5	0.000	0.000
8	AAL Toxin TE2	C27 H49 N O9	531.33898			
9	Ebelactone B	C21 H36 O4	352.25972	-6.0	0.000	0.000
10	1-Palmitoyl-2-hydroxy-sn-glycero-3-PE or 16:0 Lyso-PE	C21 H44 N O7 P	453.2834	-5.2	0.000	0.000
11	Palythene	C14 H20 N2 O7	328.25972	-4.6	0.000	0.000
12	α-Eleostearic acid	C18 H30 O2	278.22341	-5.8	0.000	0.000
13	1-O-Palmitoyl Glucuronic acid	C22 H42 O7	418.29125	-5.7	0.000	0.000
14	2-Arachidonoyl glycerol	C23 H38 O4	378.27522	-4.7	0.000	0.000
15	Eicosapentaenoic acid (C20:5, ω-3)	C20 H30 O2	302.22341	-5.3	0.000	0.000
16	Farnesylacetone	C18 H30 O	262.22878	-5.4	0.000	0.000
17	1-linoleoyl glycerol	C21 H38 O4	354.27538	-5.5	0.000	0.000
18	Kaurenoic acid	C20H30O2	303.23068	-6.5	0.000	0.000
19	Halocynthiaxanthin 3-acetate	C42 H56 O5	640.40978			

20	α-monopalmitin	C19 H38 O4	330.27559	-5.1	0.000	0.000
21	9-octadecenoic acid methyl ester	C19 H36 O3	312.26531	-4.5	0.000	0.000
22	2-Monoolein	C21 H40 O4	356.29119	-5.4	0.000	0.000
23	Glycidyl oleate	C21 H38 O3	338.28063	-4.6	0.000	0.000
24	D-glucitol monooleate	C24 H46 O7	446.32237	-5.0	0.000	0.000
25	Eicosapentaenoic acid methyl ester	C21 H32 O2	316.239	-6.2	0.000	0.000
26	Octadecatrienoic acid	C19 H32 O2	292.23891	-5.1	0.000	0.000
27	Farneside	C24 H40 N2 O8	484.27764	-6.1	0.000	0.000
28	Podecdysone B	C27 H42 O6	462.29588	-4.4	0.000	0.000
29	Protoporphyrin	C32 H34 N4 O4	538.25542			
30	Arachidonic acid	C20 H32 O2	304.23885	-6.0	0.000	0.000
31	Ponasterone A	C27 H44 O6	464.31169	-5.6	0.000	0.000
32	Pheophorbide A	C35 H36 N4 O5	592.26671			
33	Methyl pheophorbide a	C36 H38 N4 O5	606.28185			

Supplementary Table S4.

Docking results of kaurenoic acid with MC1R. The grey cells indicate the excluded ligands due to their molecular weight which exceeds 500 Dalton

No	Putative compound	Formula	Calc. MW	Binding Affinity (kcal/mol)-MC1R	Dist from rmsd l.b.	best mode rmsd u.b.
1	Acetylcholine	C7 H15 N O2	145.10977	-3.5	0.000	0.000

2	Adenine	C5 H5 N5	135.05416	-4.6	0.000	0.000
3	13S-hydroxyoctadecatrienoic	C18 H30 O3	294.21837	-3.6	0.000	0.000
4	1-(9Z-hexadecenoyl)-glycero-3-phosphoethanolamine	C21 H42 N O7 P	451.26802	-5.4	0.000	0.000
5	2,3-dihydroxypropyl 12-methyltridecanoate	C17 H34 O4	302.24428	-5.0	0.000	0.000
6	Decanoic acid	C29 H50 O4 P2 S	556.2888			
7	Stearidonic acid	C18 H28 O2	276.20784	-5.7	0.000	0.000
8	AAL Toxin TE2	C27 H49 N O9	531.33898			
9	Ebelactone B	C21 H36 O4	352.25972	-6.4	0.000	0.000
10	1-Palmitoyl-2-hydroxy-sn-glycero-3-PE or 16:0 Lyso-PE	C21 H44 N O7 P	453.2834	-4.9	0.000	0.000
11	Palythene	C14 H20 N2 O7	328.25972	-5.0	0.000	0.000
12	α-Eleostearic acid	C18 H30 O2	278.22341	-6.2	0.000	0.000
13	1-O-Palmitoyl Glucuronic acid	C22 H42 O7	418.29125	-5.8	0.000	0.000
14	2-Arachidonoyl glycerol	C23 H38 O4	378.27522	-6.1	0.000	0.000
15	Eicosapentaenoic acid (C20:5, ω-3)	C20 H30 O2	302.22341	-5.9	0.000	0.000
16	Farnesylacetone	C18 H30 O	262.22878	-6.3	0.000	0.000
17	1-linoleoyl glycerol	C21 H38 O4	354.27538	-5.5	0.000	0.000
18	Kaurenoic acid	C20H30O2	303.23068	-8.6	0.000	0.000

19	Halocynthiaxanthin 3-acetate	C42 H56 O5	640.40978			
20	α-monopalmitin	C19 H38 O4	330.27559	-4.7	0.000	0.000
21	9-octadecenoic acid methyl ester	C19 H36 O3	312.26531	-4.9	0.000	0.000
22	2-Monoolein	C21 H40 O4	356.29119	-5.1	0.000	0.000
23	Glycidyl oleate	C21 H38 O3	338.28063	-5.5	0.000	0.000
24	D-glucitol monooleate	C24 H46 O7	446.32237	-5.4	0.000	0.000
25	Eicosapentaenoic acid methyl ester	C21 H32 O2	316.239	-5.6	0.000	0.000
26	Octadecatrienoic acid	C19 H32 O2	292.23891	-5.3	0.000	0.000
27	Farneside	C24 H40 N2 O8	484.27764	-6.8	0.000	0.000
28	Podecdysone B	C27 H42 O6	462.29588	-7.2	0.000	0.000
29	Protoporphyrin	C32 H34 N4 O4	538.25542			
30	Arachidonic acid	C20 H32 O2	304.23885	-6.0	0.000	0.000
31	Ponasterone A	C27 H44 O6	464.31169	-7.1	0.000	0.000
32	Pheophorbide A	C35 H36 N4 O5	592.26671			
33	Methyl pheophorbide a	C36 H38 N4 O5	606.28185			

Supplementary References

1. Wessler, I.; Kilbinger, H.; Bittinger, F.; Kirkpatrick, C.J. The Non-neuronal Cholinergic System The Biological Role of Non-neuronal Acetylcholine in Plants and Humans. *The Japanese Journal of Pharmacology* **2001**, *85*, 2-10.
2. Lin, T.-Y.; Hassid, W.Z. Isolation of guanosine diphosphate uronic acids from a marine brown alga, Fucus gardneri Silva. *Journal of Biological Chemistry* **1966**, *241*, 3283-3293.
3. Barbosa, M.; Valentão, P.; Andrade, P.B. Biologically active oxylipins from enzymatic and nonenzymatic routes in macroalgae. *Marine drugs* **2016**, *14*, 23.
4. Lobasso, S.; Pérez-Davó, A.; Vitale, R.; Monteoliva-Sánchez, M.; Corcelli, A. Deciphering archaeal glycolipids of an extremely halophilic archaeon of the genus Halobellus by MALDI-TOF/MS. *Chemistry and Physics of Lipids* **2015**, *186*, 1-8.

5. Nurkolis, F.; Yusuf, V.M.; Yusuf, M.; Kusuma, R.J.; Gunawan, W.B.; Hendra, I.W.; Radu, S.; Taslim, N.A.; Mayulu, N.; Sabrina, N. Metabolomic Profiling, In Vitro Antioxidant and Cytotoxicity Properties of Caulerpa racemosa: Functional Food of the Future from Algae. **2022**.
6. Martyasari, N.W.R.; Ardiana, N.; Ilhami, B.T.K.; Padmi, H.; Abidin, A.S.; Sunarwidhi, A.L.; Sunarpi, H.; Nikmatullah, A.; Widyastuti, S.; Prasedya, E.S. The effect of extraction solvent polarity on cytotoxic properties of Sargassum crassifolium against B16-F10 melanoma cancer cell model. 2021; p. 012105.
7. Ishihara, K.; Murata, M.; KaneniwA, M.; Saito, H.; KoMatsU, W.; Shinohara, K. Purification of stearidonic acid (18: 4 (n-3)) and hexadecatetraenoic acid (16: 4 (n-3)) from algal fatty acid with lipase and medium pressure liquid chromatography. *Bioscience, biotechnology, and biochemistry* **2000**, *64*, 2454-2457.
8. Li, Y.; Sun, H.; Wu, T.; Fu, Y.; He, Y.; Mao, X.; Chen, F. Storage carbon metabolism of Isochrysis zhangjiangensis under different light intensities and its application for co-production of fucoxanthin and stearidonic acid. *Bioresource technology* **2019**, *282*, 94-102.
9. Wang, H.; Guo, Y.; Luo, Z.; Gao, L.; Li, R.; Zhang, Y.; Kalaji, H.M.; Qiang, S.; Chen, S. Recent Advances in Alternaria Phytotoxins: A Review of Their Occurrence, Structure, Bioactivity, and Biosynthesis. *Journal of Fungi* **2022**, *8*, 168.
10. Hashad, N.; Ibrahim, R.; Mady, M.; Abdel-Aziz, M.S.; Moharram, F.A. Bioactive metabolites and host-specific toxins from endophytic Fungi, Alternaria alternate. *Vietnam Journal of Chemistry* **2021**, *59*, 733-759.
11. Prasedya, E.S.; Syafitri, S.M.; Geraldine, B.A.F.D.; Hamdin, C.D.; Frediansyah, A.; Miyake, M.; Kobayashi, D.; Hazama, A.; Sunarpi, H. UVA Photoprotective Activity of Brown Macroalgae Sargassum Cristafolium. Biomedicines 2019, 7, 77, doi:10.3390/biomedicines7040077.
12. Monteiro, J.P.; Costa, E.; Melo, T.; Domingues, P.; Fort, A.; Domingues, M.R.; Sulpice, R. Lipidome in-depth characterization highlights the nutritional value and species-specific idiosyncrasies of different Ulva species. *Algal Research* **2022**, *64*, 102694.
13. Chen, J.; Li, M.; Yang, R.; Luo, Q.; Xu, J.; Ye, Y.; Yan, X. Profiling lipidome changes of Pyropia haitanensis in short-term response to high-temperature stress. *Journal of Applied Phycology* **2016**, *28*, 1903-1913.
14. Li, C.; Xing, X.; Qi, H.; Liu, Y.; Jian, F.; Wang, J. The arachidonic acid and its metabolism pathway play important roles for Apostichopus japonicus infected by Vibrio splendens. *Fish & Shellfish Immunology* **2022**.
15. Endo, Y.; Park, S.-B.; Fujimoto, K. Marine Conjugated Polyunsaturated Fatty Acids. In *Nutraceutical and Specialty Lipids and their Co-Products*; CRC Press: 2006; pp. 233-240.
16. Sunarwidhi, A.L.; Hernawan, A.; Frediansyah, A.; Widyastuti, S.; Martyasari, N.W.R.; Abidin, A.S.; Padmi, H.; Handayani, E.; Utami, N.W.P.; Maulana, F.A. Multivariate Analysis Revealed Ultrasonic-Assisted Extraction Improves Anti-Melanoma Activity of Non-Flavonoid Compounds in Indonesian Brown Algae Ethanol Extract. *Molecules* **2022**, *27*, 7509.
17. Susilo, B.; Rohim, A.; Wahyu, M.L. Serial Extraction Technique of Rich Antibacterial Compounds in Sargassum cristaefolium Using Different Solvents and Testing their Activity. *Current Bioactive Compounds* **2022**, *18*, 18-25.

18. Oliveira, L.S; Tschoeke, D.A; Lopes, A.C.R.M; Sudatti, D.B; Meirelles, P.M; Thompson, C.C; Pereira, R.C; Thompson, F.L. Molecular Mechanisms for Microbe Recognition and Defense by the Red Seaweed *Laurencia dendroidea. mSphere* **2017**.
19. Wakana, I. Isolation and Structural Analysis of Stimulating Substances for Morphogenesis in Marine Green Alga, Monostroma oxyspermum. *北海道大學理學部海藻研究所歐文報告* **1993**, *9*, 93-110.
20. Mevers, E.; Matainaho, T.; Allara, M.; Di Marzo, V.; Gerwick, W.H. Mooreamide A: A cannabinomimetic lipid from the marine cyanobacterium Moorea bouillonii. *Lipids* **2014**, *49*, 1127-1132.
21. Khotimchenko, S.V. Fatty acid composition of seven Sargassum species. *Phytochemistry* **1991**, *30*, 2639-2641.
22. Chen, Z.; Xu, Y.; Liu, T.; Zhang, L.; Liu, H.; Guan, H. Comparative studies on the characteristic fatty acid profiles of four different Chinese medicinal Sargassum seaweeds by GC-MS and chemometrics. *Marine drugs* **2016**, *14*, 68.
23. Debbarma, J.; Rao, B.M.; Murthy, L.N.; Mathew, S.; Venkateshwarlu, G.; Ravishankar, C.N. Nutritional profiling of the edible seaweeds Gracilaria edulis, Ulva lactuca and Sargassum sp. *Indian J. Fish* **2016**, *63*, 81-87.
24. Wang, R.; Wang, Y.; Tang, X. Identification of the toxic compounds produced by Sargassum thunbergii to red tide microalgae. *Chinese Journal of Oceanology and Limnology* **2012**, *30*, 778-785.
25. Ryu, G.; Park, S.H.; Kim, E.S.; Choi, B.W.; Ryu, S.Y.; Lee, B.H. Cholinesterase inhibitory activity of two farnesylacetone derivatives from the brown algaSargassum sagamianum. *Archives of pharmacal research* **2003**, *26*, 796-799.
26. Park, B.-G.; Kwon, S.-C.; Park, G.-M.; Ham, J.; Shin, W.-S.; Lee, S. Vasodilatation effect of farnesylacetones, active constituents of Sargassum siliquastrum, on the basilar and carotid arteries of rabbits. *Bioorganic & medicinal chemistry letters* **2008**, *18*, 6324-6326.
27. Shin, W.-S.; Oh, S.; An, S.-W.; Park, G.-M.; Kwon, D.; Ham, J.; Lee, S.; Park, B.-G. 5E- and 5Z-farnesylacetones from Sargassum siliquastrum as novel selective L-type calcium channel blockers. *Vascular Pharmacology* **2013**, *58*, 299-306.
28. Oku, N.; Hayashi, S.; Yamaguchi, Y.; Takenaka, H.; Igarashi, Y. Nostochopcerol, a new antibacterial monoacylglycerol from the edible cyanobacterium Nostochopsis lobatus. *Beilstein Archives* **2022**, *2022*, 84.
29. de Oliveira, L.S.; Tschoeke, D.A.; Magalhães Lopes, A.C.R.; Sudatti, D.B.; Meirelles, P.M.; Thompson, C.C.; Pereira, R.C.; Thompson, F.L. Molecular mechanisms for microbe recognition and defense by the red seaweed Laurencia dendroidea. *Msphere* **2017**, *2*, e00094-00017.
30. Matsuno, T.; Ookubo, M.; Nishizawa, T.; Shimizu, I. Carotenoids of sea squirts. I. New marine carotenoids, halocynthiaxanthin and mytiloxanthinone from Halocynthia roretzi. *Chemical and pharmaceutical bulletin* **1984**, *32*, 4309-4315.
31. Maoka, T.; Akimoto, N.; Murakoshi, M.; Sugiyama, K.; Nishino, H. Carotenoids in clams, Ruditapes philippinarum and Meretrix petechialis. *Journal of agricultural and food chemistry* **2010**, *58*, 5784-5788.
32. Maoka, T. Carotenoid Metabolism in Aquatic Animals. In *Carotenoids: Biosynthetic and Biofunctional Approaches*; Springer: 2021; pp. 29-49.
33. Araki, S.; Sakurai, T.; Kawaguchi, A.; Murata, N. Positional distribution of fatty acids in glycerolipids of the marine red alga, Porphyra yezoensis. *Plant and cell physiology* **1987**, *28*, 761-766.

34. Guo, Y.; Yuan, Z.; Xu, J.; Wang, Z.; Yuan, T.; Zhou, W.; Xu, J.; Liang, C.; Xu, H.; Liu, S. Metabolic acclimation mechanism in microalgae developed for CO2 capture from industrial flue gas. *Algal research* **2017**, *26*, 225-233.
35. Yu, C.-C.; Chen, H.-W.; Chen, M.-J.; Chang, Y.-C.; Chien, S.-C.; Kuo, Y.-H.; Yang, F.-L.; Wu, S.-H.; Chen, J.; Yu, H.-H. Chemical composition and bioactivities of the marine alga Isochrysis galbana from Taiwan. *Natural Product Communications* **2010**, *5*, 1934578X1000501222.
36. Prasedya, E.S.; Martyasari, N.W.R.; Abidin, A.S.; Pebriani, S.A.; Ilhami, B.T.K.; Frediansyah, A.; Sunarwidhi, A.L.; Widyastuti, S.; Sunarpi, H. Macroalgae Sargassum cristaefolium extract inhibits proinflammatory cytokine expression in BALB/C Mice. *Scientifica* **2020**, *2020*.
37. Ali, I.; Manzoor, Z.; Koo, J.-E.; Moon, S.-R.; Byeon, S.-H.; Yoo, E.-S.; Kang, H.-K.; Hyun, J.-W.; Lee, N.-H.; Koh, Y.-S. Monoolein, isolated from Ishige sinicola, inhibits lipopolysaccharide-induced inflammatory response by attenuating mitogen-activated protein kinase and NF-κB pathways. *Food science and biotechnology* **2017**, *26*, 507-511.
38. Yadaw, R.K.; Shahi, S.K. Fatty Acid Profiling Through Gas Chromatography Mass Spectrophotometry (GC-MS) of Chlorella vulgaris as Potential Feedstock for Biofuel Production. *Plant Arch* **2020**, *20*, 6039-6045.
39. Coffey, S. *Aliphatic Compounds: Penta-and Higher Polyhydric Alcohols; Their Oxidation Products and Derivatives; Saccharides: A Modern Comprehensive Treatise*; Elsevier: 2016.
40. Li, X.; Liu, J.; Chen, G.; Zhang, J.; Wang, C.; Liu, B. Extraction and purification of eicosapentaenoic acid and docosahexaenoic acid from microalgae: A critical review. *Algal Research* **2019**, *43*, 101619.
41. Yongmanitchai, W.; Ward, O.P. Screening of algae for potential alternative sources of eicosapentaenoic acid. *Phytochemistry* **1991**, *30*, 2963-2967.
42. Wu, D.; He, Y. Potential of spectroscopic techniques and chemometric analysis for rapid measurement of docosahexaenoic acid and eicosapentaenoic acid in algal oil. *Food chemistry* **2014**, *158*, 93-100.
43. Hamberg, M. Metabolism of 6, 9, 12-octadecatrienoic acid in the red alga Lithothamnion corallioides: mechanism of formation of a conjugated tetraene fatty acid. *Biochemical and biophysical research communications* **1992**, *188*, 1220-1227.
44. Gerwick, W.H.; Moghaddam, M.; Hamberg, M. Oxylipin metabolism in the red alga Gracilariopsis lemaneiformis: mechanism of formation of vicinal dihydroxy fatty acids. *Archives of biochemistry and biophysics* **1991**, *290*, 436-444.
45. Blunt, J.W.; Copp, B.R.; Keyzers, R.A.; Munro, M.H.G.; Prinsep, M.R. Marine natural products. *Natural Product Reports* **2015**, *32*, 116-211.
46. Li, Y.; Fu, X.; Duan, D.; Liu, X.; Xu, J.; Gao, X.J.M.d. Extraction and identification of phlorotannins from the brown alga, Sargassum fusiforme (Harvey) Setchell. **2017**, *15*, 49.
47. Kim, S.O.; Choi, Y.H. Indole-6-Carboxaldehyde Isolated from Sargassum thunbergii (Mertens) Kuntze Prevents Oxidative Stress-Induced Cellular Damage in V79-4 Chinese Hamster Lung Fibroblasts through the Activation of the Nrf2/HO-1 Signaling Pathway. *Cellular Physiology and Biochemistry: International Journal of Experimental Cellular Physiology, Biochemistry, and Pharmacology* **2020**, *54*, 959-974.
48. Murbach, T.S.; Glávits, R.; Moghadam Maragheh, N.; Endres, J.R.; Hirka, G.; Goodman, R.E.; Lu, G.; Vértesi, A.; Béres, E.; Pasics Szakonyiné, I. Evaluation of the

genotoxic potential of protoporphyrin IX and the safety of a protoporphyrin IX-rich algal biomass. *Journal of Applied Toxicology* **2022**.

49. Ikekawa, N.; Fujimoto, Y.; Ishiguro, M. Reminiscences of research on the chemistry and biology of natural sterols in insects, plants and humans. *Proceedings of the Japan Academy, Series B* **2013**, *89*, 349-369.
50. Lee, H.; Park, H.-Y.; Jeong, T.-S. Pheophorbide a derivatives exert antiwrinkle effects on UVB-induced skin aging in human fibroblasts. *Life* **2021**, *11*, 147.
51. Lee, J.S.; Han, J.S. Sargassum sagamianum Extract Protects INS-1 Pancreatic β-cells Against Glucotoxicity-induced Apoptosis. *한국식품영양과학회 학술대회발표집* **2017**, 302-302.
52. Giriwono, P.E.; Iskandriati, D.; Tan, C.P.; Andarwulan, N. In-vitro anti-inflammatory activity, free radical (DPPH) scavenging, and ferric reducing ability (FRAP) of Sargassum cristaefolium lipid-soluble fraction and putative identification of bioactive compounds using UHPLC-ESI-ORBITRAP-MS/MS. *Food Research International* **2020**, *137*, 109702.
53. Saide, A.; Lauritano, C.; Ianora, A. Pheophorbide a: State of the Art. *Marine Drugs* **2020**, *18*, 257.
